# Socio‐ecological drivers of vertebrate biodiversity and human‐animal interfaces across an urban landscape

**DOI:** 10.1111/gcb.15412

**Published:** 2020-12-01

**Authors:** James M. Hassell, Judy M. Bettridge, Melissa J. Ward, Allan Ogendo, Titus Imboma, Dishon Muloi, Francesco Fava, Timothy P. Robinson, Michael Begon, Eric M. Fèvre

**Affiliations:** ^1^ Institute of Infection, Veterinary and Ecological Sciences University of Liverpool Liverpool UK; ^2^ International Livestock Research Institute Nairobi Kenya; ^3^ Global Health Program Smithsonian Conservation Biology Institute Washington DC USA; ^4^ Natural Resources Institute University of Greenwich Greenwich UK; ^5^ Centre for Immunity, Infection and Evolution University of Edinburgh Edinburgh UK; ^6^ Nuffield Department of Clinical Medicine University of Oxford John Radcliffe Hospital Oxford UK; ^7^ National Museums of Kenya Nairobi Kenya; ^8^ Usher Institute of Population Health Sciences & Informatics University of Edinburgh Edinburgh UK; ^9^ Food and Agriculture Organization of the United Nations Rome Italy

**Keywords:** biodiversity, community ecology, disease, land use change, sustainable development, tropics, urbanization, wildlife–livestock–human interface

## Abstract

Urbanization can have profound impacts on the distributional ecology of wildlife and livestock, with implications for biodiversity conservation, ecosystem services and human health. A wealth of studies have assessed biotic responses to urbanization in North America and Europe, but there is little empirical evidence that directly links human activities to urban biodiversity in the tropics. Results from a large‐scale field study conducted in Nairobi, Kenya, are used to explore the impact of human activities on the biodiversity of wildlife and livestock with which humans co‐exist across the city. The structure of sympatric wildlife, livestock and human populations are characterized using unsupervised machine learning, and statistical modelling is used to relate compositional variation in these communities to socio‐ecological drivers occurring across the city. By characterizing landscape‐scale drivers acting on these interfaces, we demonstrate that socioeconomics, elevation and subsequent changes in habitat have measurable impacts upon the diversity, density and species assemblage of wildlife, livestock and humans. Restructuring of wildlife and livestock assemblages (both in terms of species diversity and composition) has important implications for the emergence of novel diseases at urban interfaces, and we therefore use our results to generate a set of testable hypotheses that explore the influence of urban change on microbial communities. These results provide novel insight into the impact of urbanization on biodiversity in the tropics. An understanding of associations between urban processes and the structure of human and animal populations is required to link urban development to conservation efforts and risks posed by disease emergence to human health, ultimately informing sustainable urban development policy.

## Graphical abstract

We live in a rapidly urbanizing world. Today, more than half of the human race lives in cities, and this number is set to continue to increase, as people migrate to urban areas in search of higher standards of living. Careful planning is required to ensure that detrimental impacts of urban development on the environment and human health are identified and addressed. In this study we use Nairobi—one of the world's fastest growing cities—as a case study with which to explore how and why people's co‐existence with wildlife and livestock varies across a rapidly developing urban landscape. By providing a detailed characterization of the patterns of human and animal co‐existence that emerge from urban socioeconomic and environmental change, and hypotheses for how this influences disease transmission, our results provide a framework within which continuing research on the ecological and epidemiological consequences of rapid urban development in the tropics can be framed.

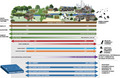

## 1 INTRODUCTION

It is generally accepted that urbanization can have widespread effects on biodiversity and health. Cities in low‐middle income countries, which are characterized by rapid, unplanned urbanization, are thought to be particularly at risk (Alirol et al., 2011). This is especially true of urban and peri‐urban zones in Africa, where growth and migration is expected to result in an increase in the population residing in these areas from 35% in 2007, to 51% by 2030 (United Nations, 2014). Urban green spaces can provide crucial ecosystem services and refugia for biodiversity, but without adequate planning, fast rates of urban growth result in substantive unplanned ecological change whilst having knock‐on effects on provision and delivery of healthcare, sanitation, demographics, trade, economics and food production (Werner, 2011).

Fragmentation of natural habitat in urban environments leads to changes in trophic structure and loss of native wildlife species, which can impact ecosystem services and human quality of life (Goddard et al., 2010). Non‐native species are frequently introduced to urban ecosystems, from which they can disperse to surrounding landscapes to the detriment of native biodiversity and ecosystem services (Gaertner et al., 2017), while also adapting to better occupy these disturbed ecological niches (Winchell et al., 2016). Variation in habitat and resource provision also presents opportunities for wildlife species that can co‐exist with humans (termed ‘synanthropes’) to thrive, resulting in homogenization of biodiversity which can lead to accelerated transmission of wildlife disease (Lowry et al., 2012). When associated with the socioeconomic characteristics of developing urban centres—social disparity (and resulting health inequalities), large‐scale migration, poor living conditions and close contact with domestic animals—such ecological changes pose a risk to human health and wellbeing through the emergence and spread of infectious disease, and lost benefits of biodiversity to immunological and mental health (Alirol et al., 2011; Hanski et al., 2012).

Land shortages, particularly in informal settlements where population growth and density are highest, mean that livestock are commonly kept within household compounds (Hassell, Ward, Muloi, Bettridge, Robinson, et al., 2019; Schiere & van der Hoek, 2001), where poor management of livestock and human waste products can contaminate the environment, and provide resources that attract urban wildlife. Urban synanthropes may act as hosts for important bacterial, viral and parasitic pathogens of humans, and mobile genetic elements conferring resistance to antimicrobials (Hassell, Ward, Muloi, Bettridge, Phan, et al., 2019) (reviewed in, Gortazar et al., 2016). Since wild and domestic animals are a key source of emerging diseases (Woolhouse & Gowtage‐Sequeria, 2005), direct interactions between these species and humans—at what are considered wildlife–livestock–human interfaces—present broad opportunities for pathogen transmission and disease emergence across urban landscapes (Hassell et al., 2017). For those responsible for mitigating the occurrence of disease emergence in urban settings to be aware of the importance of these interfaces, the risks that they pose to human health must be understood.

Consequently, urban landscapes are increasingly viewed as socio‐ecological constructs for the purposes of urban environmental and epidemiological research, in which socioeconomic and geophysical factors drive vegetation structure and the presence of resources (such as livestock and human waste), dictating wildlife species richness and abundance, and thereby the biodiversity with which humans co‐exist (Alberti et al., 2020; Des Roches et al., 2020; Kinzig et al., 2005; Leong et al., 2018; Schell et al., 2020). Although the impact of socioeconomics on plant and animal diversity and urban greenness (known as ‘the luxury effect’) has been documented in cities across the world, few studies have characterized urban biodiversity (wildlife and agricultural) in the tropics (Hope et al., 2003; Leong et al., 2018). Lack of knowledge on baseline relationships between human social organization, urban environmental change and vertebrate biodiversity currently hinder our ability to quantify the evolutionary mechanisms by which socio‐ecological change modulates native biodiversity and invasive species, whilst impacting human health and ecosystem services, across urban landscapes.

Here, we adopt a landscape ecology approach to study the socio‐ecological determinants of the vertebrate biodiversity with which humans co‐exist across Nairobi, which is one of the world's largest and most rapidly developing urban centres. As a city in the tropics, Nairobi has an inherently high biodiversity (compared, say, to European or North American cities). Our approach addresses two questions: (a) what are the city‐wide characteristics of vertebrate biodiversity (wildlife and livestock assemblages)? and (b) what is the influence of socio‐ecological drivers (variation in socioeconomics, topography and habitat) on vertebrate biodiversity within urban habitat patches? Following Lambert and Donihue (2020), Goddard et al. (2010) and research referenced within, we chose household compounds—people's houses and private land—as representative habitat patches for urban biodiversity, key sites for urban biodiversity management and important human–animal interfaces (Daniels & Kirkpatrick, 2006; Evans et al., 2009; Hassell, Ward, Muloi, Bettridge, Phan, et al., 2019). Since socio‐ecological drivers operate at a broader scale than the individual household and most wildlife species are not constrained to household limits, we consider the influence of local and landscape‐scale drivers on the interface between vertebrate biodiversity and humans by sampling triplets of adjacent households (99 in total) grouped by socioeconomic status across the city.

## 2 MATRIALS AND METHODS

### 2.1 Study design and data collection

This section provides a brief summary of study design and data collection (a detailed explanation is provided in Methodological Appendix S1). A stratified sampling design was used in this study, for which Nairobi was split into administrative sublocations (70 of these administrative subunits make up Metropolitan Nairobi), and 33 were chosen on the basis of socioeconomic stratification into seven wealth groups as part of the UrbanZoo project (Bettridge et al., 2017). The number of sublocations assigned to each wealth group was chosen proportionately to the population density and the variety of neighbourhood classes (categorized by physical landscape attributes and demographic details) in each of the seven wealth groups. Final selection of sublocations was aimed at maximizing areas with high livestock densities, whilst ensuring coverage of wealth groups and geographical distribution. For each sublocation, three households were randomly selected (a total of ninety‐nine households), with the aim of maximizing the spatial distribution and diversity of socioeconomic conditions, urban habitats and livestock‐keeping practices captured within the sampling frame. Households in each sublocation had to meet strict inclusion criteria of keeping either small ruminants or poultry, large ruminants or pigs or no livestock within the household compound. The combination of livestock‐keeping households represented in each sublocation was randomized, and had to consist of two households keeping either large ruminant or poultry, or large monogastric or small ruminant species. Characterization of household land use, wildlife and livestock populations and socioeconomic indicators is described in the following paragraphs.

Land use, comprising natural and artificial habitats, was classified for each household compound. Visual classification of habitat types within each household compound and a 30 m buffer area were conducted consistently at a 1:500 scale on a 1 m resolution ESRI World Imagery satellite‐image available in ArcGIS 10.5 (ESRI, imagery captured in 2017) (see Methodological Appendix S1). Nine natural and artificial habitat types were visually identified and represented as proportions of total household area; waterbody, wetland, crops, mature trees, shrubs, grassland, bare ground, artificial ground (any man‐made surface) and rubbish. Natural habitats (all except bare ground, artificial and rubbish) were used to calculate Simpson's index of diversity for ‘living’ (biotic) habitats available to wildlife within households, ranging from 1 (maximum heterogeneity) to 0 (one habitat type only). Topography was measured as elevation and determined using the Google Maps API Elevation Service. Visual classification was subsequently ground‐truthed by revisiting sites.

Cross‐sectional data were collected on the presence of avian species, and select mammal taxa (rodents, fruit bats, insectivorous bats, non‐human primates (NHPs) and small carnivores) in each household compound, from biological sampling activities, ecological surveys and the household questionnaire (see Methodological Appendix S1). Avian and mammalian taxa were grouped into ecologically relevant functional groups, by their feeding and positional ecology, using the EltonTraits database (Wilman et al., 2014; Table 1). For birds, the number of different species in each functional group was also calculated in each household. Wildlife biodiversity was estimated from the presence of wildlife species/functional groups within each household. Since we were unable to establish a reliable method of surveying the presence of mammalian species within households, we relied on more easily identifiable mammalian functional groups as a proxy for the diversity of mammals present in each household environment. Wildlife diversity (β‐diversity—diversity within communities) was approximated by adding avian species richness (the total number of avian species recorded in a household) to the number of mammalian functional groups identified as being present in each household.

**Table 1:** Functional groups by which wildlife species were grouped according to their feeding and (for birds) positional ecology. Primates and carnivores were each considered as a single functional group

Avian functional groups	Rodent functional groups	Bat functional groups
Feeding ecology—strata	Feeding ecology	Feeding ecology
Plant/seed‐eating—low canopy	Omnivore	Insectivorous
Plant/seed‐eating—high canopy		Fruit
Omnivorous—low canopy		
Omnivorous—high canopy		
Fruit/nectar‐eating—low canopy		
Fruit/nectar‐eating—high canopy		
Invertebrate‐eating—low canopy		
Invertebrate‐eating—high canopy		
Vertebrate/fish‐eating/scavenger—low canopy		
Vertebrate/fish‐eating/scavenger—high canopy		

The socioeconomic status and characteristics of human and livestock populations within each household were derived from questionnaires detailing human occupants, their assets and livestock ownership and management (see Methodological Appendix S1). Wealth and ruralness indices for each household were calculated based on methods used to create the Demographic and Health Surveys (DHS) wealth index, which is derived from a Principal Component Analysis (PCA) of easily measurable household assets (such as access to water, construction materials and ownership of livestock). Dividing human and livestock abundance by household area (m^2^, as measured using ArcGIS) generated an estimate of density of livestock and humans within each habitat patch. Each human participant in the study (members of the household who consented to take part in the study, *n* = 293) completed a separate questionnaire, detailing their level of education.

### 2.2 Structure of wildlife–livestock–human interfaces

Self‐organizing maps (SOMs), a form of unsupervised machine learning that behave in a similar way to clustering algorithms, were used to explore the co‐occurrence of wildlife and livestock species and therefore describe the composition of urban vertebrate communities. SOMs are particularly useful for creating spatially organized representation of data and discovering correlation in multivariate datasets.

Two SOMs were constructed in the R package ‘kohonen’ (Wehrens & Buydens, 2007) to describe (a) relationships between vertebrate communities and broader characteristics of the human and livestock populations with which they co‐exist, and (a) co‐occurrence of all wildlife functional groups and livestock species. Each map consisted of 7 × 8 nodes, with each node representing an array of values corresponding to the input variables. Input variables for the first SOM were wildlife and livestock diversity, livestock density and human density, while input variables for the second SOM were community datasets for presence/absence of wildlife functional groups and livestock. To identify the contribution of each input category to variance between SOM nodes (and thus clustering of the data), a Bayesian approach to feature significance was used (features being variables such as wildlife diversity). In this, the probability of each feature (e.g. variable such as wildlife α‐diversity) capturing the structure of the data was compared within a probabilistic framework in the R package ‘popsom’ (Hamel & Brown, 2012).

### 2.3 Associations between urban socio‐ecological drivers, habitat and vertebrate biodiversity

To determine how socio‐ecological drivers (including human social constructs and different forms of habitat structure) affect the structure of wildlife and livestock communities across Nairobi, statistical models were used to test four hypotheses. Determinants for habitat structure and the form of wildlife and livestock communities were considered separately, testing whether (a) socioeconomic and environmental drivers (wealth, education and topography) influence the structure of urban habitats and wildlife diversity, (b) environmental divers (topography), natural and artificial habitats and anthropogenic resource provisioning influence wildlife assemblages, and (c) social determinants (e.g. wealth and education) influence livestock‐keeping practices. Our final hypothesis tested whether the compositional distinctiveness of vertebrate biodiversity (how distinct the wildlife and livestock assemblages in a single habitat patch are in relation to others) was associated with changes in urban land use.

Six response datasets were represented in these models. Wildlife diversity (avian species richness and the number of mammalian functional groups per household) was considered as a single variable. Proportions of household habitat types were considered as a single dataset. Household vertebrate biodiversity was split into four community datasets; one with binary presence/absence of all wildlife functional groups per household (*n* = 99), one with abundance of avian functional groups per household (*n* = 99), one with abundance of livestock per household (*n* = 66) and one combining presence/absence of wildlife functional groups with abundance of livestock per household (*n* = 66). A Hellinger transformation was applied to the first three of these community datasets, to account for heterogeneity in animal detection probabilities (Legendre & Gallagher, 2001). The wildlife–livestock dataset was transformed into a distance matrix, with the Jaccard dissimilarity index representing dissimilarity (β‐diversity—diversity between ecological communities) in wildlife–livestock community composition between households. Local contributions to β‐diversity (LCBD) indices, derived by decomposing the total β‐diversity represented in a community dataset (BD_total_) into site and species‐based contributions (Legendre & De Cáceres, 2013), were used as a measure of the compositional distinctiveness of wildlife–livestock communities in relation to one another. A single LCBD value was calculated for each household LCBD from the Jaccard dissimilarity index using the function *betadiv* in the R package ‘adespatial’ (Dray et al., 2017). Spatial structure in each response dataset was represented and controlled for using distance‐based Moran's eigenvector maps (dbMEMs), which provide a powerful multivariate approach to model spatial structure in a response variable, and can be partitioned by broad, medium and fine spatial scales (see the Statistical Appendix S1 for further details) (Borcard et al., 2004). dbMEM eigenvectors modelling significant spatial variation in each response dataset were included as partial terms, thus removing spatial variation from the model.

Multivariate response datasets (proportions of household habitat, and wildlife and livestock community datasets) were regressed against explanatory variables using canonical redundancy analysis (RDA; models *Habitat*, *Wildlife* ^1^, *Wildlife* ^2^, *Avian* ^1^, *Avian* ^2^ and *Livestock* in Table 2). Correlation between artificial land use and tree cover resulted in specification of two candidate models for the wildlife and avian community datasets. Variance partitioning was used for *Wildlife* and *Avian* models, to separate variation by sets of explanatory variables—anthropogenic (un‐natural habitats and resource provisioning) and ecological (natural habitat) factors, both anthropogenic and ecological factors and an unexplained component (Figure S1; Borcard et al., 1992). Statistical significance of each fraction with respect to all others was tested using RDA and analysis of variance (ANOVA). For the *Habitat* and *Livestock* models, partial RDAs were used, permitting the presence of significant spatial structure in the response variable (represented as dbMEM eigenvectors) to be controlled for. All forms of canonical analysis were computed in the R package ‘vegan’ (Okansen et al., 2017). Univariate response variables (wildlife diversity and household LCBD values) were regressed against explanatory variables using mixed effects models (Table 2). The *Wildlife diversity* model was fitted with a *Poisson* distribution, and the LCBD model was fitted with a linear distribution using the R package lme4 (Bates et al., 2017).

**Table 2:** *R* ^2^ values for global and optimal RDA and mixed effects models (GLMM/LMM), and F‐statistics/coefficients for variables included in these models. Variables in green and blue represent ‘ecological’ and ‘anthropogenic’ drivers/habitat features respectively. *F*‐statistics are depicted for statistically significant variables (*p* < .05) in RDA models (red tiles), and coefficients are depicted for statistically significant variables (*p* < .05) in mixed effects models (blue tiles). ^1^ denotes model including tree cover (not artificial land use); ^2^ denotes model including artificial land use (and not tree cover); * (grey) means that the variable was included in the global model only, and not after selection for the optimal model; * (red) means that the variable was included in the optimal model, but was not statistically significant

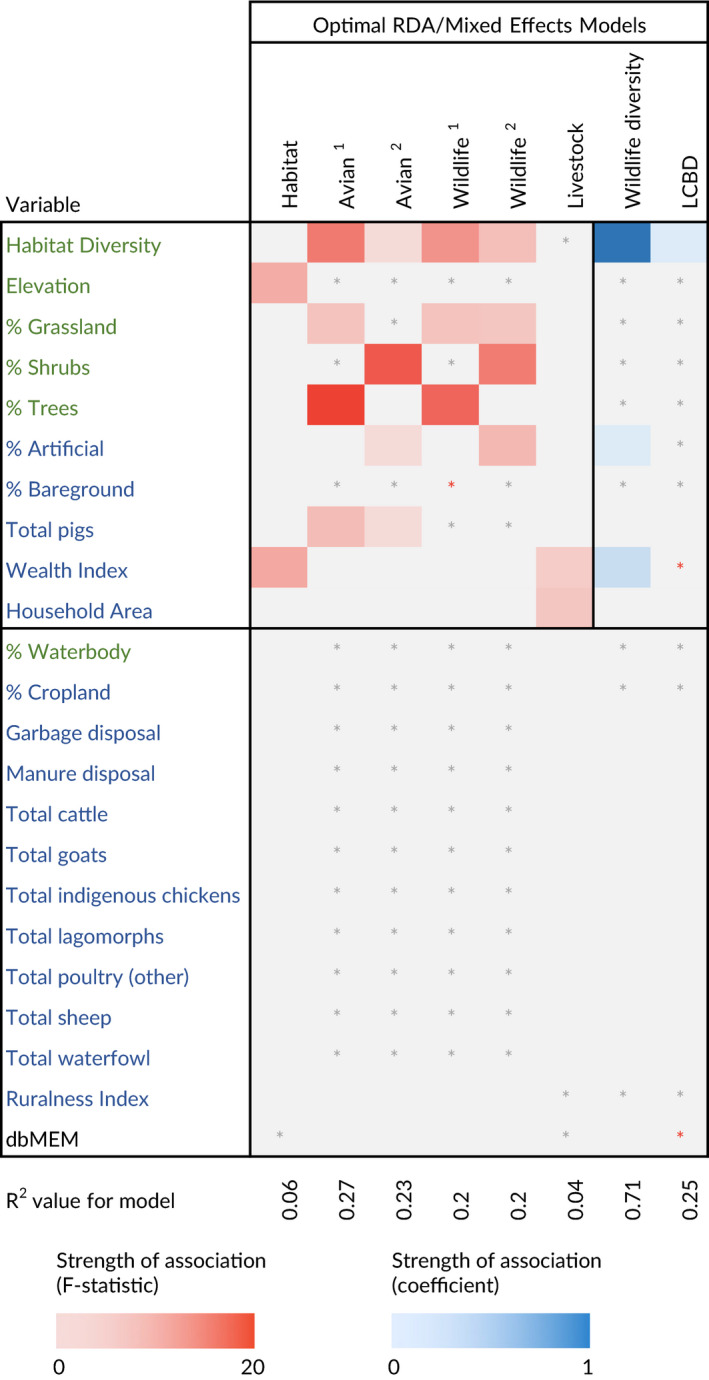

In all models, household dependency in the sampling design was accounted for by constraining permutations within sublocations (canonical models) or including sublocation as a random effect (mixed models). The explanatory variables education and wealth were highly correlated, meaning that only wealth was included in models. Optimal canonical models were chosen using forward selection with double stopping criteria, implemented with the *ordiR2step* function in R package ‘vegan’ (Blanchet et al., 2008). Optimal mixed models were chosen using stepwise, backwards elimination from the full model based upon Akaike information criteria (AIC). Significance of model terms were tested by 999 permutations or maximum likelihood test for canonical and mixed models respectively, and the fit of each model was reported as regression coefficients of multiple determination (*R* ^2^_adj_) for canonical models or marginal R^2^ for mixed models. All canonical and mixed models (full candidates and their optimal derivatives) are depicted in Table 2. Data exploration and model validation procedures were carried out as described by Zuur et al. (2010).

## 3 RESULTS

### 3.1 Variation in vertebrate biodiversity

**Figure 1:**
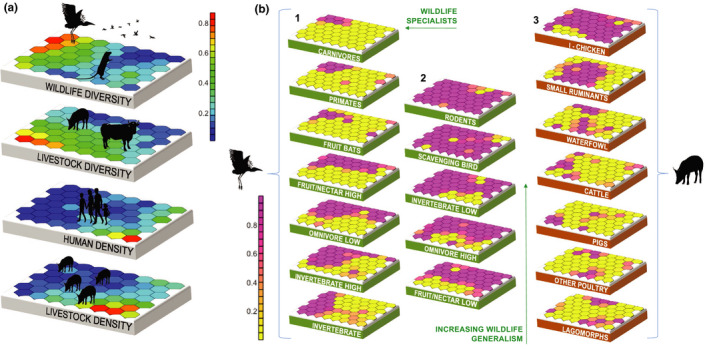
Self‐organized maps (SOMs) as applied to broad‐ and fine‐scale community characteristics in households. (a) SOM component planes for household variables (wildlife diversity, livestock diversity, human density and livestock density) included in the broad‐scale model. Each 7 × 8 grid represents the data for that variable projected into a two‐dimensional space that is common across all four grids. Similar samples (in this case households) are mapped more closely together, and coloured by vector value (blue = low, red = high). For example, the resulting maps show that households with high human and livestock density tend to have low wildlife diversity. (b) SOM component planes for each variable in the fine‐scale models [the presence of wildlife functional groups (green planes; panel 1 = ‘specialist’ wildlife functional groups, panel 2 = ‘generalist’ wildlife functional groups) and the presence of livestock species (brown planes, panel 3)]. High and low refer to canopy strata detailed in Table 1. Each 7 × 8 grid represents the data for that variable projected into a two‐dimensional space that is common across all 19 grids. Samples (in this case households) with similar values for each variable are mapped more closely together, and coloured by vector value (yellow = absence, pink = presence). The distribution of different wildlife functional groups across SOM component planes (and therefore households) are used to indicate whether they are ‘specialists’ or ‘generalists’ with regards to habitat utilization

We used self‐organizing maps (SOMs) to explore co‐occurrence of wildlife and livestock species and therefore describe the composition of urban vertebrate communities in 99 households across the city. At a broad scale, variation in household assemblages arose mainly from wildlife diversity (Bayesian feature significance: wildlife diversity, 0.683; livestock diversity, 0.305; livestock density, 0.008, human density, 0.004), while household densities of humans and livestock were highly correlated with one another and negatively correlated with wildlife diversity (Figure 1a). At a finer scale, species‐level structure of household interfaces showed clear patterns of distributional overlap within and between wildlife and livestock, enabling identification of frequently co‐occurring taxa, which could be categorized into ‘generalists’ (those widely distributed across the urban landscape, and frequently co‐occurring), and ‘specialists’ (wildlife constrained to ecological niches, or livestock kept according to anthropogenic determinants) (Figure 1b, panels 1–3). Synanthropic species (rodents, scavenging and seed‐eating birds and insectivorous bats) were found ubiquitously in households, and as such frequently co‐occurred with commonly kept livestock species (indigenous chickens and small ruminants), generating ‘baseline’ wildlife–livestock interfaces, with which humans co‐exist. Households with higher densities of humans and livestock were associated with the presence of generalist wildlife taxa (particularly rodents and scavenging birds) and chickens.

### 3.2 Associations between urban socio‐ecological drivers, habitat and vertebrate biodiversity

#### 3.2.1 Habitat

Proportions of different habitat type (natural and artificial) were regressed against socioeconomic and environmental drivers (wealth, education and topography) using canonical redundancy analysis (RDA), to determine how socio‐ecological drivers impact land use. The structure of household habitats was predicted by wealth and elevation (*R* ^2^_adj_ = .06; Table 2). Wealthier households were correlated with greater proportions of tree cover and lower proportions of bare ground, and higher elevations were correlated with greater proportions of tree cover and lower proportions of grassland (Figure S2).

#### 3.2.2 Wildlife diversity

**Figure 2:**
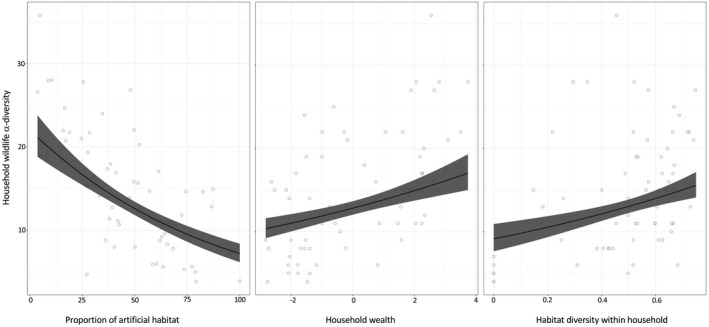
Plots for the effects of landscape‐scale drivers on household biodiversity (wildlife). Shaded line represents fit of the generalized linear mixed effects model, demonstrating the effect of proportions of artificial habitat, wealth and habitat diversity on β‐diversity of wildlife within household compounds. For each plot, all other covariates in the model are kept constant

Three datasets were used to explore the response of urban wildlife diversity to socioeconomic and environmental drivers, and variation in habitat and resource provision. Since we were unable to measure species diversity of mammals within each household, household wildlife diversity was approximated by combining richness of avian species and mammalian functional groups in household environments (Table 1). Wildlife diversity was regressed against socioeconomic and environmental drivers (habitat diversity, household wealth, education and topography) within a *Poisson*‐distributed generalized linear mixed effects model. Increasing wildlife diversity was associated with higher wealth and habitat diversity (measured as Simpson's index of diversity for natural habitats available to wildlife within household compounds) and lower proportions of artificial land use (model *Wildlife diversity*; *R* ^2^_adj_ = .7; Table 2; Figure 2).

RDA models were used to regress community datasets representing the presence/absence of wildlife functional groups, and abundance of avian groups per household (*n* = 99), against environmental drivers (topography) and a detailed set of household‐level ecological and anthropogenic covariates capturing variation in natural and artificial habitat and resource provisioning (livestock‐keeping, and manure/garbage disposal practices). Correlation between artificial land use and tree cover resulted in specification of two candidate models for each response dataset. Wildlife functional group assemblages were best explained by habitat diversity, proportions of grassland and tree cover (model *Wildlife* ^1^: *R* ^2^_adj_ = .201) or habitat diversity and proportions of grassland, shrubs and artificial land use (model *Wildlife* ^2^: *R* ^2^_adj_ = .197; Table 2). Relationships were evident between variables: the presence of primates, carnivores and fruit bats (urban specialists) was positively correlated with tree cover, and negatively correlated with proportions of artificial land use (Figure S3). Communities dominated by rodents and insectivorous bats (urban generalists) were positively correlated with artificial land use, and negatively correlated with ecological variables (trees, grassland, shrubs and habitat diversity). Variance partitioning within the RDAs showed that ecological factors explained all variation in wildlife functional groups for model *Wildlife* ^1^ (0|0.201), whilst ecological and anthropogenic factors explained similar variance in community structure in model *Wildlife* ^2^ (0.149|0.129; Table 2).

Avian community structure was best explained by habitat diversity, abundance of pigs and proportions of grassland and trees (model *Avian* ^1^: *R* ^2^_adj_ = .267), or habitat diversity, abundance of pigs, elevation and proportion of artificial land use (model *Avian* ^2^: *R* ^2^_adj_ = .23; Table 2). Households with lower proportions of shrubs and tree cover, more homogenous biotic habitats and higher proportions of artificial land use were more likely to have species assemblages dominated by scavenging and low‐strata seed‐eating birds (generalists), whilst households with more trees, diverse biotic habitats and shrubs were positively correlated with omnivorous, invertebrate‐eating and fruit/nectar birds occupying high and low strata (specialists; Figure S4). Both low‐strata invertebrate‐eating birds and generalist invertebrate‐eating birds were associated with higher abundances of pigs. Variance partitioning showed that ecological factors accounted for significantly larger proportions of variance in avian community structure in both models (*Avian* ^1^: 0.02|0.267, *Avian* ^2^: 0.126|0.159; Table 2).

#### 3.2.3 Livestock diversity

Determinants for the species of livestock being kept across the city were explored by regressing a community dataset comprising the abundance of livestock species kept within household compounds (*n* = 66) against socioeconomic indices, household area and biotic habitat diversity of households in an RDA. Variation in livestock assemblages was associated with household wealth and area (model *Livestock*: *R* ^2^_adj_ = .04; Table 2).

#### 3.2.4 Compositional distinctiveness of household biodiversity

Finally, we examined how urbanization influences stability of the animal communities with which people co‐exist, by regressing a measure for the compositional distinctiveness of each household's vertebrate biodiversity—local contributions to β‐diversity (LCBD)—against a set of anthropogenic and ecological variables selected to represent possible determinants for both wildlife and livestock diversity in households, in a linear mixed effects model. LCBD values are strictly positive, and increase as the community of potential hosts at each site becomes more unique; sites with large LCBD values could therefore represent urban ecological conditions in which potential host community structures depart from normality (Legendre, 2014).

Household LCBD values were associated with changes in habitat diversity, wealth and the spatial eigenvector MEM10 (representing spatial variation across medium spatial scales; model *LCBD*: marginal *R* ^2^ = .253, Table 2). Habitat diversity was the only statistically significant term in this model, and was negatively correlated with LCBD (*β* = −0.006, 95% CI = −0.009 to −0.002, *p* < .001; Figure S5). As habitats become less ecologically complex, LCBD increases and vertebrate assemblages become more distinct.

## 4 DISCUSSION

**Figure 3:**
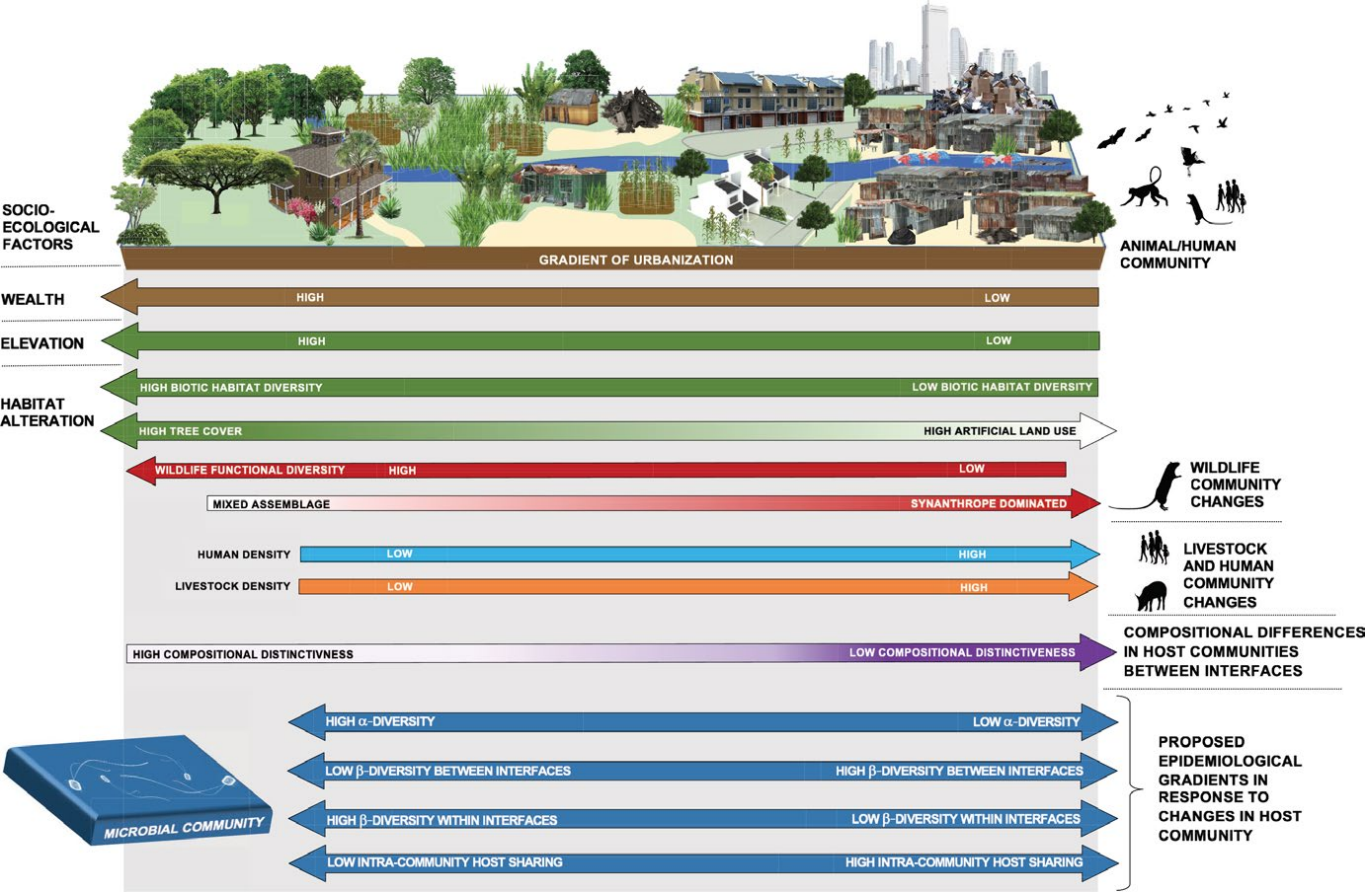
Diagrammatic representation of the impact of socio‐ecological drivers on habitat and vertebrate biodiversity along the urban gradient. Landscape‐scale drivers are labelled on the left, and the responses of different animal and human assemblages to variation in these drivers are labelled on the right. This schematic brings together the main results from this study, presenting a unified picture of the socio‐ecological effects of urban development on household animal–human interfaces. The bottom panel considers hypothetical epidemiological responses of microbial communities to changes in host community dynamics brought about by urban land‐use change

The social structures of human society play a tremendous role in transforming urban environments from natural to artificial ecological states (Leong et al., 2018). In this study, methods from community ecology were applied to describe how rapid urbanization influences the ecological conditions (habitat and assemblages of wildlife and livestock) that humans experience in Nairobi. The results reveal variation across multiple levels of urban biological organization, enabling us to establish characterizations for animal–human interfaces that exist along gradients of urbanization, and identify drivers that have contributed to their formation (Figure 3).

### 4.1 Biodiversity along an urban ecological gradient

Nairobi, like other urban environments in the tropics, is characterized by high heterogeneity of land use, resulting from fragmentation of biologically diverse natural habitats through anthropogenic activities. Change from ecological (biotic) to predominantly anthropogenic (abiotic) habitats has a profound impact on the community assemblage of wildlife species in common with other cities (Gibb et al., 2020; Lowry et al., 2012). Linking household wildlife assemblages to habitat structure, we have shown that as the intensity of anthropogenic habitats (artificial land use) increases, functionally diverse communities of birds and mammals that utilize restricted niches (such as frugivores, nectarivores and primates) are replaced by urban generalists (rodents, scavenging and seed‐eating birds and insectivorous bats), capable of utilizing resources in a broad variety of environmental and anthropogenic niches.

Those households lying at the ‘anthropogenic extent’ of urban land use that support wildlife communities of low species and functional diversity dominated by urban synanthropes co‐exist with high densities of humans, and high density, low or medium diversity livestock communities, characterized by indigenous poultry, pigs and small ruminants (Figure 3). At the opposite end of the scale (and on the periphery of the city) household sites are ecologically closer to their preurbanized state, featuring diverse biotic habitats, populated by progressively more functionally complex wildlife communities which co‐exist with low densities of humans and their livestock. The distinctiveness of wildlife–livestock communities (LCBD indices) was negatively associated with biotic habitat diversity, indicating that interfaces were more unusual at the anthropogenic end of the urban habitat spectrum. This is at odds with other studies, that have identified a trend towards ‘biotic homogenization’ (biodiversity becoming more homogenous) under increasing levels of urbanization (McKinney, 2006). This observation could be explained by the extreme variation in types of urban development seen in developing cities such as Nairobi—for example, the most densely populated settlements in the city are located along riparian areas, and on the edge of forests harbouring high levels of biodiversity (Bagnis et al., 2020; Furukawa et al., 2011). This juxtaposition of natural and artificial habitats argues against over‐simplifying the concept of urbanization, which is often described as occurring along a linear gradient.

### 4.2 Socio‐ecological drivers of vertebrate biodiversity and animal–human interfaces

Elevation and socioeconomics were identified as important determinants for habitat structure and the wildlife diversity with which humans co‐exist in Nairobi. Elevation (ranging from 1,484 to 1,936 metres above sea level), which was a significant predictor of tree cover, represents a broader set of geophysical factors that reflect the preexisting Afromontane forest and savannah biomes within which the city is located. Nairobi's more affluent neighbourhoods have historically been located at higher altitudes, and in common with other studies (Hope et al., 2003) we detected a strong luxury effect, where wealthier households had greater proportions of forested habitat and biodiversity. However, wealth accounted for significantly more variance in habitat structure than elevation alone, which suggests that current socioeconomic trends occurring outside the city's historical pattern of land use, such as a rapidly growing middle‐class with the disposable income to manage their land, are influencing habitat structure and biodiversity.

Wealth, education and city governance would also be expected to influence the supply of resources available to wildlife through agricultural crops, livestock, their products, and some evidence suggests that these factors affect the distributional ecology of urban wildlife (reviewed in Becker et al. (2015)). With the exception of a significant correlation between the abundance of insectivorous birds and households keeping pigs, we were unable to detect associations between the presence of crops, livestock or their waste and wildlife assemblages. Variation in arthropod numbers associated with pig‐keeping could account for higher abundances of insectivorous birds in the presence of pigs. Coprophagous insects (and larvae) presumably thrive in the presence of pigs, which are predominantly kept in informal settlements in Nairobi in low‐biosecurity conditions (Alarcon et al., 2017). Without measuring abundances of mammals and birds which would have increased the resolution and discriminatory power of our analyses, this dataset may lack the resolution to detect these patterns more widely (Barwell et al., 2015). Since resource provisioning is known to generate novel assemblages of species (Galbraith et al., 2015), impact wildlife health (Murray et al., 2018) and promote transmission of disease (recently described in this study system Hassell, Ward, Muloi, Bettridge, Phan, et al., 2019; Hassell, Ward, Muloi, Bettridge, Robinson, et al., 2019), the effects of urban livestock‐keeping on the distributional ecology of wildlife deserves further investigation.

Livestock are commonly kept for cultural reasons and as a source of food security in developing cities, where human population growth outpaces the growth in provision of services, employment and food accessibility. Correlations between livestock assemblages, wealth and area suggest that the availability of resources required to sustain different species dictate people's livestock‐keeping practices. Less resource‐specific species with a broader diet (pigs, goats and indigenous chickens) tend to be favoured by low‐income and/or space‐limited households, whilst species that rely on more restricted diet (cattle, rabbits, sheep) are kept by more affluent households and/or those with more space.

### 4.3 Implications for urban development policy

Understanding the eco‐evolutionary dynamics of urbanization is paramount to developing sustainable urban management strategies (Alberti et al., 2020; Des Roches et al., 2020), and we provide an illustration of the patterns that emerge from the intersection between biological and sociological complexity in Nairobi. Our findings therefore have important implications for the sustainable planning and management of cities, particularly rapidly developing, biodiverse cities. Our results reinforce the importance of maintaining patches of diverse natural habitats (particularly forest and grassland) to increase wildlife biodiversity. Promoting habitat diversity is also important for avoiding the formation of novel urban ecosystems (NUE’s, represented as artificial habitats in this study), which we found support more distinct assemblages of wildlife. Kowarik et al. (2011) describe how conversion to built‐up areas or heavily changed urban land that represent NUEs can facilitate invasion and onward spread of alien species and affect ecosystem services. Until more evidence on the impacts of exotic plants in a tropical urban context is available, urban developers should be careful to ensure that appropriate habitats, representing the preexisting biomes in which cities are built, are established to support native species.

Provision of access to urban green space and the ecosystem services it provides is one of the United Nations’ key development goals for 2030 (United Nations, 2015), but our results demonstrate that predetermined socioeconomic barriers restrict access to vertebrate biodiversity for urban citizens in Nairobi. This follows a general pattern in cities worldwide, where wealth segregation shapes the ecological structure of urban ecosystems (Schell et al., 2020). Gardens in particular play a crucial role for maintaining, and ensuring people have access to, urban biodiversity (Goddard et al., 2010). As such, increasing per capita GDP and development programs to upgrade slums and raise income levels in many developing countries presents a unique opportunity to expand and redistribute urban green space, and full advantage should be taken to increase and re‐establish diverse habitats at the most altered end of the urban continuum. Not only could this improve individual health and prosperity—non‐communicable diseases such as atopic dermatitis (Hanski et al., 2012; Ruokolainen et al., 2015), inflammatory bowel disease (IBD) (Cholapranee & Ananthakrishnan, 2016) and psychological conditions (Fuller et al., 2007) have been associated with lower biodiversity of urban environmental conditions, and people who live near parks typically enjoy higher property values—but it could also mitigate broader environmental challenges facing urban environments in the tropics, which are at higher risk from anthropogenic climate change. Habitat restoration in heavily developed urban areas could compensate higher‐intensity rainfall, ambient temperatures and rates of evaporation and higher levels of pollution associated with urban growth. Given the precipitous biodiversity loss in the tropics, and with many African cities set to double in size within the next thirty years, green spaces will provide an increasingly important link between biodiversity and citizens, and should therefore be prioritized for biodiversity conservation.

The distribution and density of animal hosts are critical components of zoonotic disease transmission, and spillover of novel pathogens into people (Fenton & Pedersen, 2005; Plowright et al., 2017). As such, through compositional changes in microbial communities, the urban restructuring of wildlife and livestock assemblages observed in this study has important implications for human health and wellbeing in rapidly urbanizing settings (Roche et al., 2012). Taking two broadly accepted principals of microbial‐host community dynamics and relating them to variation in vertebrate biodiversity described in this study, ecological and epidemiological urban trends in microbial dynamics can be inferred and used to generate a set of testable hypotheses that would improve our understanding of the epidemiological consequences of urban land‐use change (Figure 3). These hypotheses follow the assumption that host and microbial diversity are correlated (the exact nature of this relationship would depend upon host specificity—saturation is expected to occur more quickly when communities are dominated by microbial parasites with low host specificity (Ostfeld & Keesing, 2017)), and changes in host relative abundance and density influence the compositional stability of the microbial community (Hanski et al., 2012; Roche et al., 2012). Recently published work from this study system in Nairobi has demonstrated that communities of bacterial genes encoding virulence and antimicrobial resistance are structured according to the diversity and density of co‐existing avian, livestock and human communities and the habitat within which they exist (Hassell, Ward, Muloi, Bettridge, Phan, et al., 2019).

As wildlife assemblages within households become more functionally uniform (and livestock and human density increases), major compositional changes will occur in their microbial communities. At the biotic end of the urban land‐use spectrum, microbes exist within diverse, relatively stable vertebrate communities, and as such, would be expected to belong to equally diverse and stable communities (Mosites et al., 2017; Tyakht et al., 2013). At the ‘anthropogenic’ end of the urban spectrum, microbial communities existing within a restricted host niche would be expected to be of lower diversity, while facing higher selection pressures. A recent study conducted on American white ibises ﻿(*Eudocimus albus*) demonstrated that microbial diversity was lost along a gradient of urbanization, which correlated with higher shedding of pathogenic Salmonella—suggesting that urbanization could lead to microbial perturbations that favour pathogen colonization (Murray et al., 2020). Here, we also hypothesize that microbial communities are increasingly divergent than those present at other household interfaces, as the compositional distinctness of host assemblages (measured as LCBD indices) increases. Given that interfaces at the ‘anthropogenic’ end of the spectrum tended to have higher densities of livestock and humans, and contain species that are competent hosts for a high diversity of zoonoses (rodents, bats, pigs and chickens Gibb et al., 2020; Olival et al., 2017), such conditions could present ideal circumstances for spillover and amplification of pathogens to occur. On this basis, surveillance of at‐risk human and animal populations, which is considered the most effective and cost‐effective way of combating emerging infectious diseases (Holmes et al., 2018), would be best focused in low‐income, livestock‐keeping households, composed of high densities of humans, livestock and synanthropic wildlife.

## 5 CONCLUSIONS

The effects of urbanization on public health and biodiversity have been identified as a key knowledge gap﻿ (Gibb et al., 2020). Using Nairobi as a case study, we demonstrate that socio‐ecological drivers such as wealth and elevation shape a rapidly developing urban environment and the vertebrate species within it. Such insight into the spatial organization of urban biodiversity is required to inform frameworks for urban biodiversity management, such as those recently proposed by Lambert and Donihue (2020). Through a detailed characterization of urban wildlife–livestock–human interfaces and hypotheses for how urban development influences microbial ecology, we also provide insight into how rapid urbanization can generate interfaces for pathogen emergence, which should be targeted for surveillance. These findings have important implications for urban development planning in the tropics and should be considered as evidence that can guide efforts to make Nairobi and other biodiverse cities more sustainable, healthy and environmentally balanced in the future. What is learned from our study in Nairobi where urban citizens, their livestock and wildlife live in close association, could also be applied to urban development planning in other rapidly developing cities with the aim of improving human and environmental health.

## AUTHOR'S CONTRIBUTIONS

J.H., E.M.F and T.R conceived the study. J.H. performed fieldwork, conducted data analysis and drafted the manuscript. E.M.F., J.B., M.J.W., T.R., D.M., F.F. and M.B. were involved in study design and provided comments on the manuscript. A.O. and T.I. collected the field data. All authors gave final approval for publication.

## Supporting information

Fig S1‐S5

Appendix S1

## Data Availability

Data are available via an open access repository held by the University of Liverpool (datacat.liverpool.ac.uk/470).
