# Supplementary material for: Socio‐ecological drivers of vertebrate biodiversity and human‐animal interfaces across an urban landscape
Source: Glob Chang Biol. 2020 Dec 1;27(4):781–92. doi: 10.1111/gcb.15412 (PMC7983883; doi:10.1111/gcb.15412)
Supplement: Supplementary file 1 — Fig S1‐S5 [file GCB-27-781-s002.pdf]

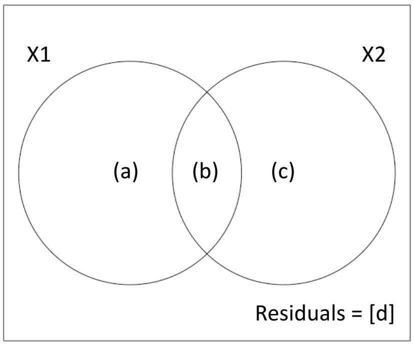

Figure S1: Variance partitioning fractions, when considering two sets of explanatory variables. Throughout this study, the circle 'X1' relates to variation derived from anthropogenic determinants, and the circle 'X2' relates to variation derived from environmental determinants. [a|b] denotes the variation explained by anthropogenic variables only, [b|c] denotes the variation explained by environmental variables only, [b] denotes the variation explained by both environmental and anthropogenic variables, and [d] denotes the variation remaining unexplained. The statistical significance of each fraction with respect to all others can be tested using redundancy analysis and ANOVA.

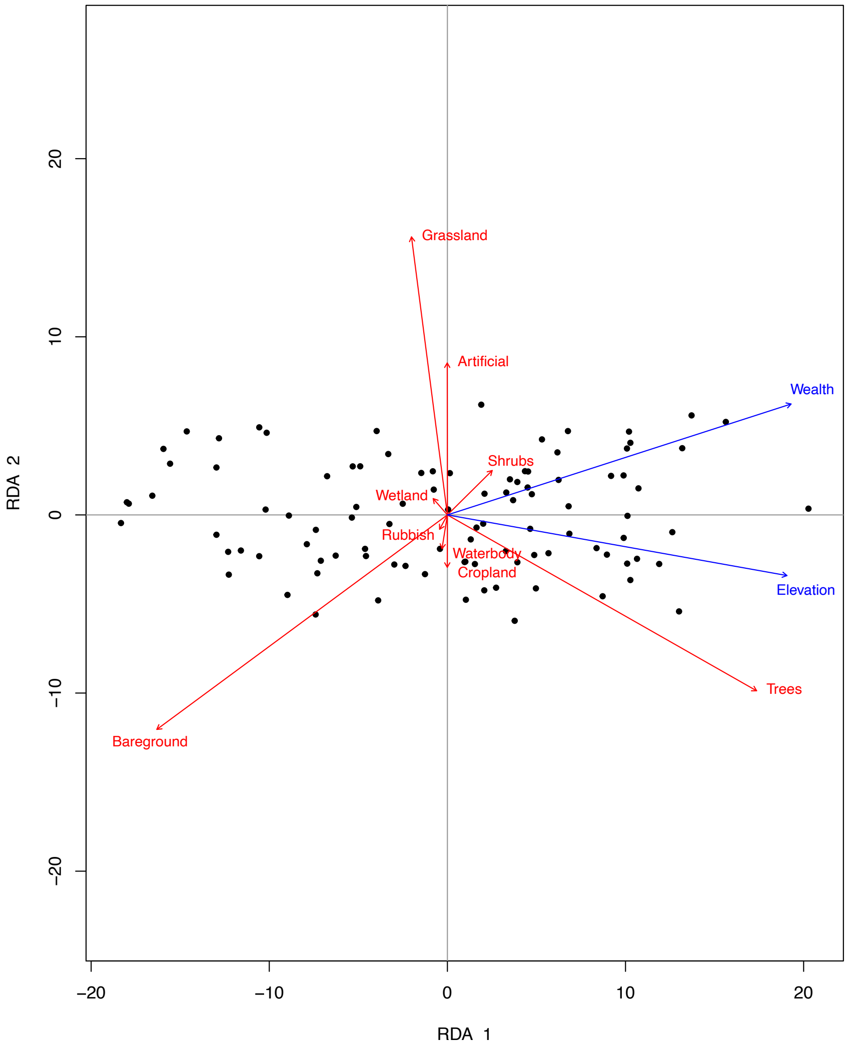

Figure S2: Correlation triplot for optimal redundancy analysis (RDA) model of habitat (model H). Grey dots represent households. Labels in red are habitat types and labels in blue are landscape-scale processes.

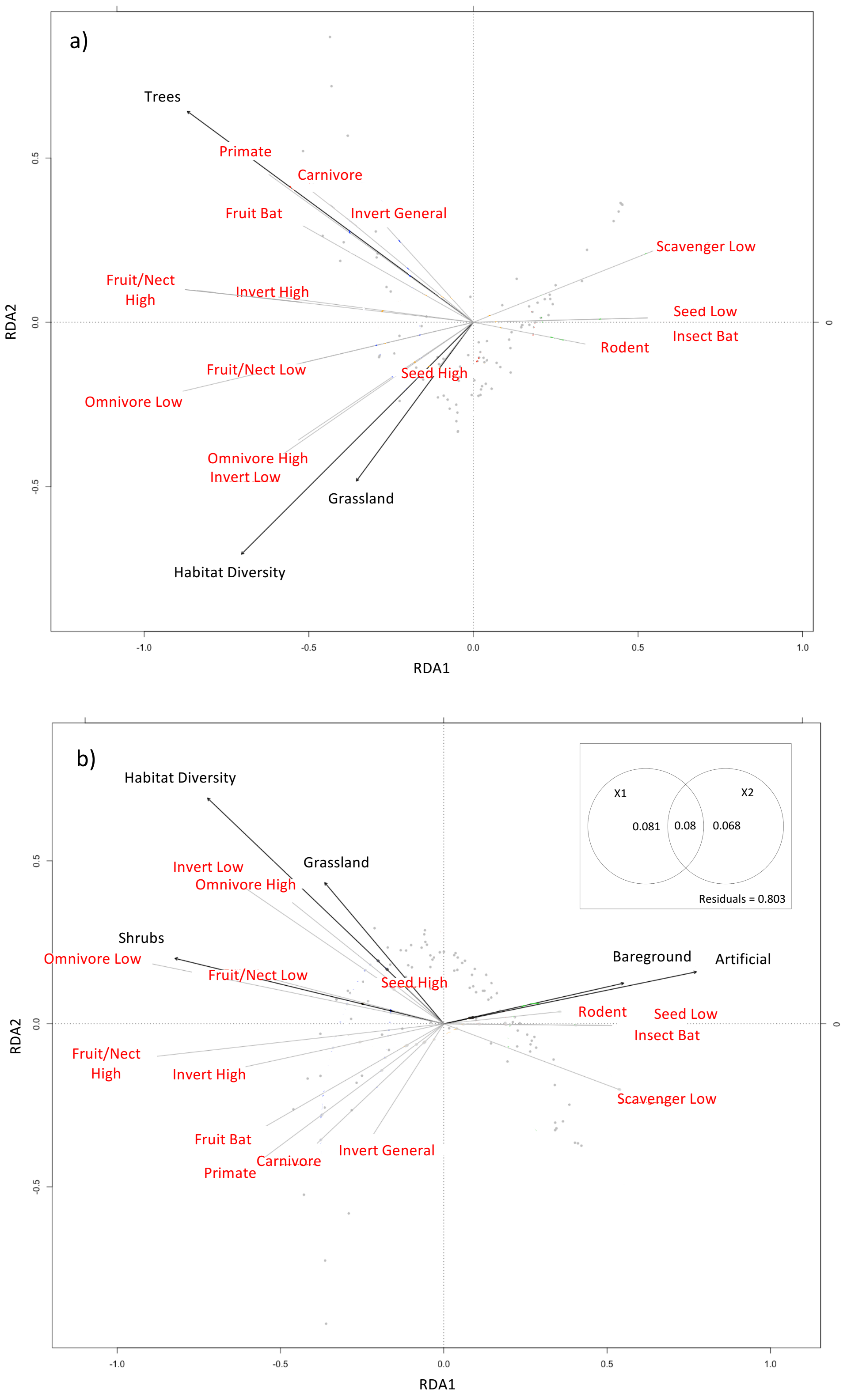

Figure S3: Correlation triplots for optimal redundancy analysis (RDA) models of the wildlife community dataset (models W^1 and W^2). a) Model including tree cover rather than artificial land use (response variables red, explanatory variables black), **b)** model including artificial land use rather than tree cover (response variables red, explanatory variables black). Grey dots represent households. Ellipses represent household modes (as determined in the broad scale self-organising map); green = mode 1, red = mode 2, orange = mode 3, blue = mode 4. Box in the upper right corner of plot **b)** indicates R^2_{adj} values for each set of explanatory variables (derived from variance partitioning), where X1 = anthropogenic determinants, and X2 = environmental determinants.

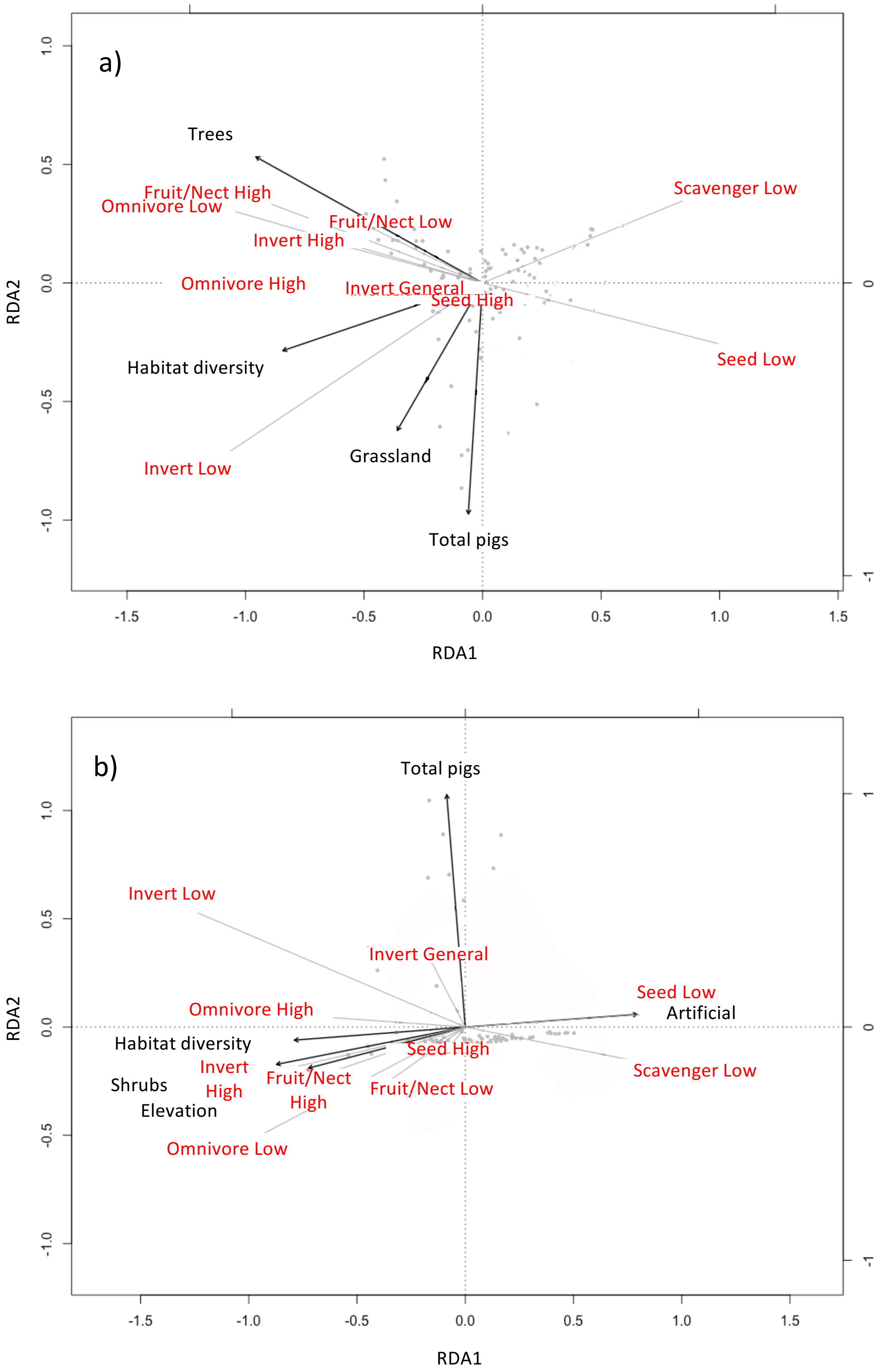

Figure S4: Correlation triplots for optimal redundancy analysis (RDA) models of the avian community dataset (models A^1 and A^2). a) Model including tree cover rather than artificial land use (response variables red, explanatory variables black), **b)** model including artificial land use rather than tree cover (response variables red, explanatory variables black). Grey dots represent households. Ellipses represent household modes (as determined in the broad scale self-organising map).

a)

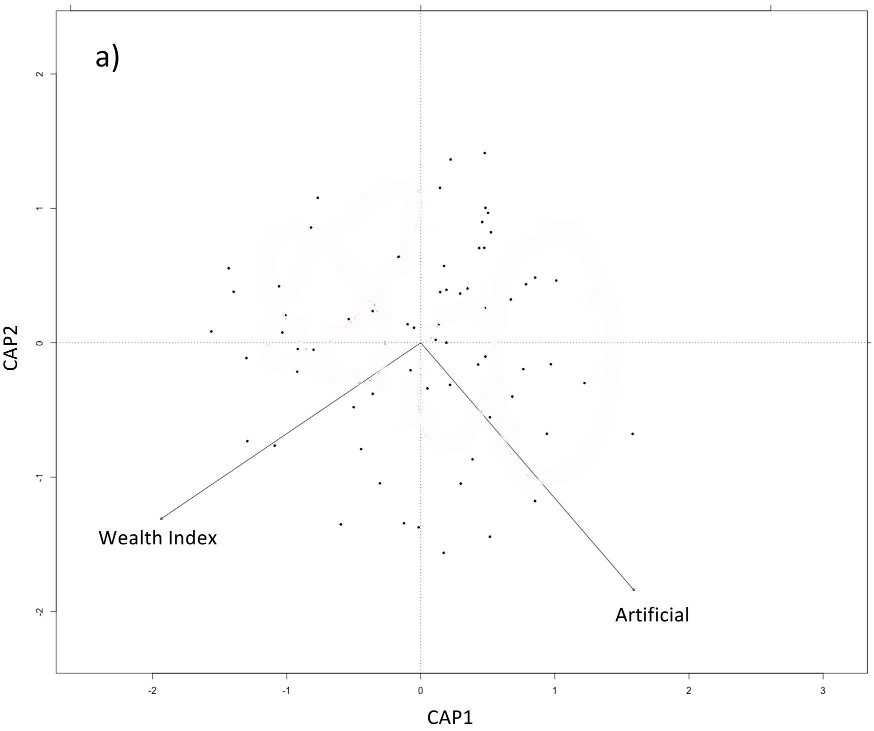

b)

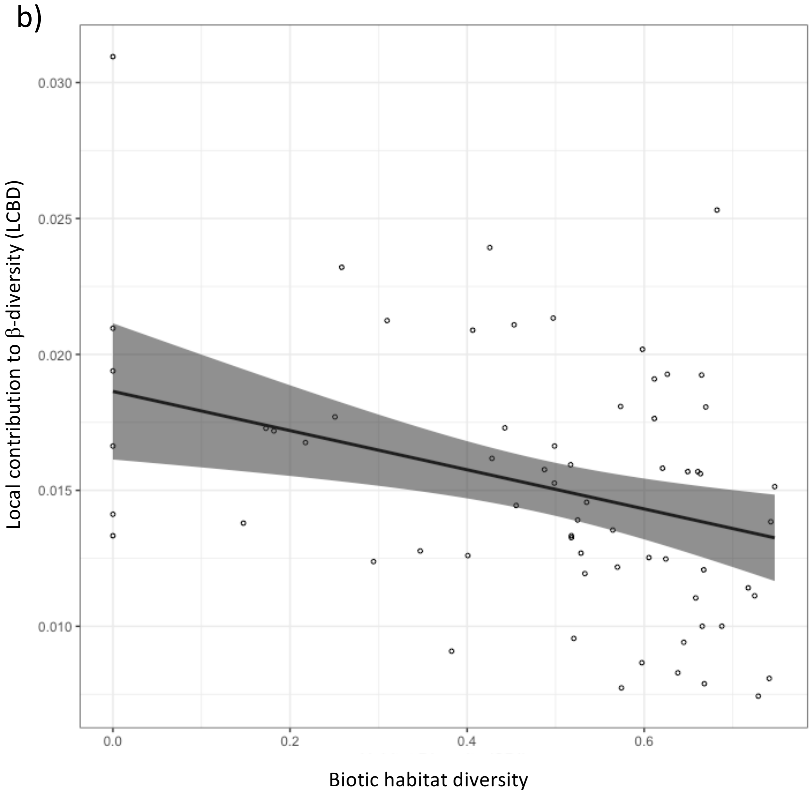

Figure S5: Plots for distance-based β-diversity analysis a) Correlation triplot of optimal distance-based redundancy analysis model for the wildlife and livestock community dataset (WL^2). Response variables (principle coordinates representing the (dis)similarities between household assemblages) are represented as black points, explanatory variables are black lines and text. Ellipses represent household modes (as determined in the broad scale self-organising map); green = mode 1, red = mode 2, orange = mode 3, blue = mode 4. **b)** Fit of the linear mixed effects model for household local contribution to β–diversity (LCBD) values, demonstrating the effect of biotic habitat diversity in households on LCBD value of households. All other covariates in the model are kept constant.
