# Supplementary material for: Socio‐ecological drivers of vertebrate biodiversity and human‐animal interfaces across an urban landscape
Source: Glob Chang Biol. 2020 Dec 1;27(4):781–92. doi: 10.1111/gcb.15412 (PMC7983883; doi:10.1111/gcb.15412)
Supplement: Supplementary file 2 — Appendix S1 [file GCB-27-781-s001.pdf]

Supplementary Methodological Appendix S1

Study Design

The UrbanZoo project, based in Nairobi, Kenya from 2012-2017, aimed to utilise a landscape genetics approach to understanding the movement and sharing of pathogens in a major developing city. A key component of this project, within which this study was nested, was the '99 household project', which focused on informal livestock keeping practices in urban households as a route of zoonotic disease emergence in humans. As such, households were selected with the aim of maximising the spatial distribution and diversity of livestock keeping practices across Nairobi, and were chosen to capture three main criteria: socio-economic diversity, population distribution and livestock keeping practices. Geospatial mapping data, generated as part of a technical report produced by Institut Français de Recherche en Afrique (IFRA), was used to identify 17 classes of residential neighbourhood in Nairobi based on physical landscape attributes, which were subsequently verified by 817 household questionnaires (Ledant, 2011). Each of the 17 classes of neighbourhood were then ranked by average income and reduced into seven wealth groups. Administrative sublocations were mapped onto each wealth group, identifying a total of 70 possible sublocations, for which dominant wealth groups were calculated by extracting the proportion of population belonging to each neighbourhood class within the sub-location boundaries (Table SM1). A total of 33 sublocations were selected to be included in the study, with the number of sublocations belonging to each wealth group chosen proportionately to the population density and the variety of neighbourhood classes in each of the seven wealth groups. Final selection of individual sublocations was aimed at maximising areas with high livestock densities, whilst ensuring coverage of other neighbourhood classes and geographical spread.

Field sampling was conducted between September 2015 and September 2016. For each sublocation, three geographical points were selected at random within the dominant housing type. The order in which sublocations were visited was randomised. Local officials assisted in the recruitment of a household closest to each geographical point, to obtain two livestock keeping and one non-livestock keeping household per sublocation (a total of 99 households, 66 of which kept livestock). The closest households to each geographic point that met the inclusion criteria were selected. Households had to meet strict inclusion criteria of

keeping either large ruminants (cattle), large monogastrics (pigs), small ruminants (goats/sheep), small monogastrics (poultry/rabbits), or no livestock species. To ensure an equal sample of both cattle and pig-keeping households, the combination of livestock keeping households represented in each sublocation was randomised, and had to consist of either large ruminant and small monogastric, or large monogastric and small ruminant species. For sublocations in which households keeping large ruminant or large monogastric species were absent, a replacement household keeping either small monogastric or small ruminant species was recruited.

Data Collection

Wildlife Trapping and Ecological Surveys. Ecological surveys were used, alongside trapping data, to estimate the diversity of wildlife species present within households. Rodents, bats, birds, small carnivores and non-human primates (NHPs) were all targeted for trapping in each household, with the intention of collecting faecal samples for the broader UrbanZoo project. Rodents were trapped using medium-sized (23 cm x 7.5 cm x 9 cm) Sherman live traps (H. B. Sherman Traps Inc., Tallahassee, FL) or Victor lethal traps (Woodstream Corp., Lititz, PA) that were baited with dried fish, placed against walls throughout the household and livestock keeping facilities, and left in place for three nights. Where possible, traps were set in each household for all trapping nights and checked daily. Mist nets were set at dawn to trap birds, with nets being positioned outside the house and around livestock keeping facilities. For household compounds in which bat activity was deemed likely (as judged based on the presence of fruiting trees and/or ‘flyways’), mist nets were set at dusk and monitored for two hours. If members of the household reported seeing small carnivores (such as mongoose) then Tomahawk cage traps (Tomahawk Live Trap Company, Tomahawk, Wis.) were set, baited with chicken and monitored regularly for a maximum of three days. Where NHP activity was reported at a household, wire-mesh live-capture traps were pre-baited with bananas for a minimum of three days. Traps were then set, and monitored regularly for a maximum of three days. Due to large variation in the size of household compounds, trapping effort (i.e. number of traps/mist nets placed per trapping session) was scaled to the size of the household compound.

Avian species counts (presence/absence) were conducted by a trained ornithologist from the National Museums of Kenya, in which species were identified based on audio-visual identification over a 20-minute period spent walking transects of each household compound. Surveys were conducted between 6:30am and 9:30am, over the course of two months in the dry season, ensuring that bird activity and weather conditions were constant. Where security conditions permitted, a remote bat detector (Song Meter ZC, Wildlife Acoustics, Inc.) was placed in each household compound for a single night, to monitor ultrasonic bat activity. All spectrograms were visually examined for bat echolocations by a trained mammologist. As part of a household questionnaire (detailed in the next section), members of the household were also asked whether they saw rodents, small carnivores (such as mongoose) and NHPs within the household compound (with the aid of pictures).

Household and Individual Human Questionnaires. A nominated member of each household completed a questionnaire, detailing *i)* livestock ownership, management, sourcing, sales and antimicrobial use, and *ii)* household composition and socio-economic data. Abundance (counts) of livestock species and humans, and manure and household waste management practices were derived from this data for each household. Household composition and socio-economic data were used to generate 'wealth' and 'ruralness' indices for each household sampled (Bettridge et al., 2017). These indices were calculated based on methods used to create the Demographic and Health Surveys (DHS) wealth index, which is derived from a Principal Component Analysis (PCA) of easily measurable households assets (such as access to water, construction materials and ownership of livestock) (Johnson & Oscar Rutstein, 2004). A modification was made to the original set of household assets included in the DHS index to better capture household variation in Nairobi. An individual questionnaire was also administered to each human participant in the 99 household study, which captured demographic information, contact with livestock, perceptions of urban wildlife, and a detailed health assessment. Level of education for each household member was recorded on a scale of 1 (lowest) to 7 (highest), and an average generated for each household. All field data was recorded using Open Data Kit (ODK) Collect software (Hartung et al., 2010), on electronic tablets, and uploaded to databases held on servers at the International Livestock Research Institute (ILRI).

Land-use classification. Nairobi is characterised by a large variety of land use. The boundary of each household compound was drawn in ArcMap, and a 30m buffer created around the perimeter of each compound to represent the landscape surrounding it. A buffer of 30m was chosen to reflect home range of common urban rodent species (*Mus* and *rattus* spp., estimates of which vary from 1m to 30m) (Jones et al., 2009; Lambert, Quy, Smith, & Cowan, 2008). Visual classification of land-use types within the compound and buffer area were conducted at 1:500 scale on a 1m resolution ESRI World Imagery satellite-image available in ArcGIS 10.5 (ESRI). Characterisation of ecological characteristics along a perimeter around the household compound was considered as important, because the ecological setting within which the household exists extends beyond the boundaries of the compound. The extent to which this influential area of habitat outside the compound extends is unknown, and as such it was standardised across study sites. Within the boundary, the areas of nine different land-use types were visually identified and sketched as polygons; water-body, wetland, crops, mature trees, shrubs, grassland, bare ground, artificial ground and rubbish (descriptions for each of these are summarised in Table SM2). The total area of classified land-use types at each site were calculated and expressed as proportions. Ecological land-use types (all except bare ground, artificial and rubbish) were used to calculate Simpson's diversity index, which considers both habitat richness, and an evenness of abundance among the land-use types present at each site. This index was created to represent the α–diversity of 'living' (biotic) habitat niches available to wildlife within households, and ranged from 1 (maximum heterogeneity) to 0 (only a single category of land use present). All classification was undertaken by J.M.H. who was familiar with the landscape at each site, and subsequently ground-truthed by revisiting sites.

Supplementary Statistical Appendix S2

Modelling Spatial Structure with dbMEMs. Urban environments and their host communities are spatially heterogeneous. Understanding spatial structures present across the urban landscape and in these communities, could indicate the underlying processes that have created them, and as such, it is important to capture this spatial structure in statistical models. In complex ecological and epidemiological systems, spatial structure operates across multiple scales, and a single response variable can display structure at more than one spatial scale

(Legendre & Gauthier, 2014). To address this, a statistical method called Distance-based Moran's eigenvector maps (dbMEM) was used to represent spatial structure across all scales in the models in this study (Borcard, Legendre, Avois-Jacquet, & Tuomisto, 2004). This approach begins with identifying the scales at which spatial structure (autocorrelation) is present in the response variable. The dbMEM base functions (eigenvectors) which represent structure in the response variable, are generated through a PCoA performed on a matrix of geographic distances between sites (households). Linear trends are tested for, and, if identified in the data, spatial detrending is applied before conducting dbMEM analysis (as recommended by Boccard et al (2004)). Eigenvectors modelling positive spatial correlation are extracted, and regressed against the response variable to determine a set of significant dbMEM base functions modelling spatial structure in the response variable. These base functions can then be included as explanatory variables in a global model, or their variance can be removed from the model if spatial structure is deemed a 'nuisance variable'. To make them ecologically meaningful, dbMEM base functions can be split into arbitrary groups representing different spatial scales. Generally, base functions can be ordered from broad- to fine-scale as their number increases (e.g. Figure SM1) (Legendre & Gauthier, 2014).

Bettridge, J. M., Robinson, T. R., Hassell, J. M., Kariuki, S., Ward, M. J., Woolhouse, M. E. J., & Fèvre, E. M. (2017). Soup-E.coli-field logistics-epi-and-genomics: A sampling strategy to capture bacterial diversity in a changing urban environment. *Society for Veterinary Epidemiology and Preventative Medicine, Inverness, March 29-31*.

Borcard, D., Legendre, P., Avois-Jacquet, C., & Tuomisto, H. (2004). Dissecting the spatial structure of ecological data at multiple scales. *Ecology*, *85*(7), 1826–1832. https://doi.org/10.1890/03-3111

Johnson, K., & Oscar Rutstein, S. (2004). The DHS Wealth Index. In *DHS Comparative Reports No. 6*. Bethesda, Md: ORC Macro.

Jones, K. E., Bielby, J., Cardillo, M., Fritz, S. A., O'Dell, J., Orme, C. D. L., … Purvis, A. (2009). PanTHERIA: a species-level database of life history, ecology, and geography of extant and recently extinct mammals. *Ecology*, *90*(9), 2648–2648. https://doi.org/10.1890/08-1494.1

Lambert, M. S., Quy, R. J., Smith, R. H., & Cowan, D. P. (2008). The effect of habitat management on home-range size and survival of rural Norway rat populations. *Journal*

of Applied Ecology, *45*(6), 1753–1761. https://doi.org/10.1111/j.1365-2664.2008.01543.x

Ledant, M. (2011). Socio-Economical and Infrastrucural Mapping and Analysis of Nairobi: Technical Report. In *IFRA/GWOPA/UN-HABITAT, Nairobi, Kenya*.

Legendre, P., & Gauthier, O. (2014). Statistical methods for temporal and space-time analysis of community composition data. *Proceedings. Biological Sciences / The Royal Society*, *281*(1778), 20132728. https://doi.org/10.1098/rspb.2013.2728

Characteristics of physical neighbourhood classes identified by IFRA study (adapted from Ledant *et al.* [1])				Urban Zoo Project re-classification		
Tree cover	Defining characteristics	Neighbourhood description (housing type)	Average income	Wealth group	Possible sub-locations	Targeted sub-locations
> 13.5%	Detached housing with intense tree cover	Detached housing on very large plots *(>3000 m²)*	39,890	1	8	3
		Detached housing on large plots *(400 - 3000 m²)*	22,462	2	8	4
	Attached and semi-detached housing	Attached housing on medium plots *(<400 m²)* with important tree cover	22,084	2		
3% < 13.5%	Apartment building	Apartment buildings *with gated space*	22,084	2		
	Attached and semi-detached housing	Higher standing row houses *(plot size > 190 m²)*	13,352	3	5	3
		Lower standing row houses *(plot size < 190 m²)*	6,153	4	3	3
<3%	Roof cover >50% tiles	Lower standing *apartment buildings*	6,153	4		
		New areas of dense *single housing* development	3,855	5	9	5
	Roof cover > 40% concrete	High density multi-storey buildings	3,855	5		
3% < 13.5%	Apartment building	Apartment buildings *with open access*	3,855	5		
	Peripheral areas	Peripheral areas with residential component *(mainly residential)*	3,855	5		
		Peripheral areas with rural component *(presence of agriculture)*	2,165	6	24	11
	Collective housing	Community housing *with gated space*	2,165	6		
		Community housing *with open access*	2,165	6		
<3%	Roof cover >85% corrugated iron sheets	New areas of low quality housing *(built-up area <37%)*	2,165	6		
		High density planned low quality housing *(built-up area <37% AND public space >20%)*	2,165	6		
		High density unplanned low quality housing (slums) *(built-up area <37% AND public space <20%)*	1,301	7	13	4

Table SM1. The seven wealth groups used by the UrbanZoo Project, and the number of sub-locations with a dominant wealth group identified and selected in the Nairobi municipality. Reprinted with permission from Bettridge *et al.* [2].

Land Use Class	Explanation
Water-body (environmental, biotic)	Natural water body (flowing or non-flowing)
Wetland (environmental, biotic)	Open wetland – reeds/rushes
Cropland (anthropogenic, biotic)	Row cops or other herbaceous crops (e.g. maize, coffee etc.)
Trees (environmental, biotic)	Trees (single or multiple), as determined by presence of a clear crown and evidence of shadow cast on ground (includes plantations of trees for commercial purposes [e.g. fruit])
Shrubs (environmental, biotic)	Shrubs, where able to distinguish from grassland and trees
Grassland (environmental, biotic)	Grass, pasture, herbaceous rangeland or bare-ground not serving a human purpose.
Bare ground (anthropogenic, abiotic)	Heavily compacted soil, serving human purpose (e.g. dirt road, playground)
Artificial (anthropogenic, abiotic)	Synthetic, man-made surface or object (including water-bodies) (e.g. tarmac road, cement, roof, swimming pool, water tank)
Rubbish (anthropogenic, abiotic)	Accumulation of human-derived waste

Table SM2: Land use classifications

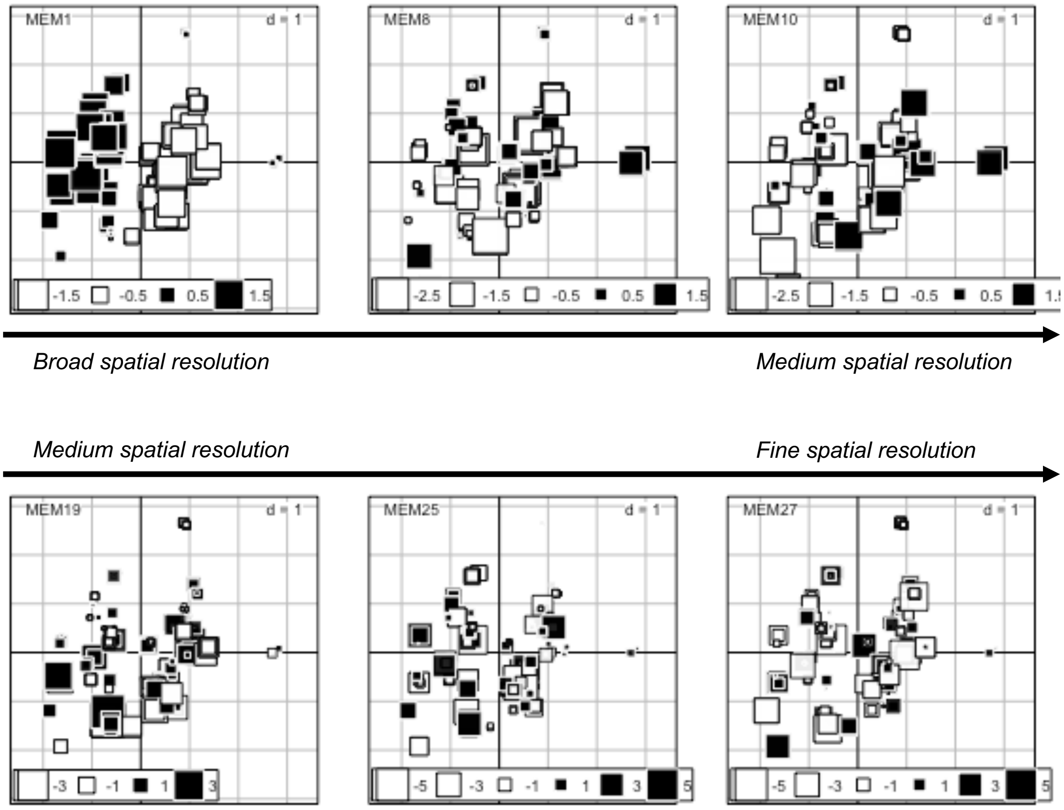

Figure SM1. Plot showing dbMEM eigenvectors modelling spatial variation in households at different scales across Nairobi. Increasing numeric magnitude of dbMEMs represents a gradient of broad to fine-scale spatial structure. As such, in this example MEM1, MEM8 and MEM10 model broad through to medium scale spatial resolution, and MEM19, MEM25 and MEM27 model medium through to fine-scale spatial resolution across Nairobi. Black and white blocks represent the GPS location of households.
